# Is flexion-extension imaging necessary for surgical decision-making in degenerative lumbar spondylolisthesis? Supine MRI and upright radiographs offer superior diagnostic value

**DOI:** 10.1016/j.bas.2026.106006

**Published:** 2026-03-06

**Authors:** Tom Folkerts, Lukas Schönnagel, Luis Bürck, Maximilian Muellner, Kirsten Labbus, Thilo Khakzad, Matthias Pumberger, Friederike Schömig

**Affiliations:** Center for Musculoskeletal Surgery, Charité – University Hospital Berlin, Berlin, Germany

**Keywords:** Degenerative lumbar spondylolisthesis, Lumbar spinal fusion, Degeneration, Spinal stability, Lumbar spine, Segmental instability

## Abstract

**Introduction:**

Degenerative lumbar spondylolisthesis (DLS) is a major indication for lumbar surgery. Surgical decision-making, particularly regarding the need for fusion, often depends on radiographic detection of segmental instability. Flexion–extension (FE) radiographs remain the reference standard but are limited by radiation exposure, motion-related discomfort, and poor reproducibility, whereas upright–supine (US) imaging - combining upright lateral radiographs with supine MRI - may offer a comparable, low-radiation alternative using routinely acquired images.

**Research question:**

To compare segmental motion at L4/5 between FE and US imaging and evaluate their respective abilities to identify patients with radiographic signs of instability.

**Material and methods:**

In this retrospective cross-sectional study, 128 patients surgically treated for isolated L4/5 DLS were included. Segmental motion was analyzed using FE and US imaging by determining relative slippage, dynamic slip angle, and radiographic instability, defined as sagittal translation ≥8% and/or a dynamic slip angle ≥10°.

**Results:**

Relative slippage was comparable between US and FE imaging (US: 7.43 ± 5.10%; FE: 7.29 ± 4.57%; p = 0.994). The dynamic slip angle was significantly higher in US imaging compared to FE imaging (US: 6.42 ± 4.20°; FE: 4.03 ± 2.98°; p < 0.001). US imaging identified more patients with radiographic signs of instability compared to FE imaging (US: n = 68, 53.1%; FE: n = 42, 32.8%; p < 0.001).

**Discussion and conclusion:**

US imaging identified a higher proportion of patients meeting established thresholds for radiographic instability compared to FE radiographs. Although FE imaging remains the diagnostic standard, US imaging may offer a practical, low-radiation alternative for preoperative assessment. Prospective investigations are warranted to confirm its clinical utility.

## Highlights

• US imaging detected more patients with radiographic instability than FE radiographs.
• Dynamic slip angle was significantly higher in US imaging than in FE imaging.
• Relative slippage was comparable between US and FE imaging.
• US imaging may provide a low-radiation approach for instability evaluation.

## List of abbreviations

ASA: American Society of Anesthesiology
BMI: Body Mass Index
CT: Computed Tomography
DLS: Degenerative Lumbar Spondylolisthesis
E: Extension
F: Flexion
FE: Flexion-Extension
ICC: Intraclass Correlation Coefficient
ILS: Isthmic Lumbar Spondylolisthesis
MRI: Magnetic Resonance Imaging
RS: Relative Slippage
S: Supine
SA: Slip Angle
U: Upright
US: Upright-Supine

## 1 Introduction

Degenerative lumbar spondylolisthesis (DLS) is defined as an acquired vertebral subluxation, characterized by the displacement of a vertebral body relative to the subjacent segment, typically in an anterior direction (Saremi et al., 2024; Matz et al., 2014; Elmose et al., 2023; Campbell et al., 2017). In contrast to isthmic lumbar spondylolisthesis (ILS), DLS occurs in the presence of an intact posterior vertebral arch and is primarily driven by progressive intervertebral disc degeneration (Rangwalla et al., 2024; Wiltse, 1981). This degenerative process alters spinal biomechanics by redistributing axial load to the facet joints, thereby promoting segmental instability, facet joint arthropathy, and ultimately leading to spinal canal stenosis, vertebral slippage, and structural deformities (Saremi et al., 2024; Rangwalla et al., 2024; Kalichman and Hunter, 2008; Lee and Patel, 2013). Most frequently involving the L4/5 segment, DLS is a leading indication for lumbar spine surgery and is often associated with low back pain, accompanied by leg pain, radiculopathy, or neurogenic claudication (Saremi et al., 2024; Denard et al., 2010; Jacobsen et al., 2007; Spiker et al., 2019; Fujimori et al., 2015).

The optimal surgical strategy for symptomatic DLS remains debated. Fusion as an adjunct to decompression is not universally endorsed, as randomized trials report comparable outcomes irrespective of whether fusion is performed (Austevoll et al., 2021; Hellum et al., 2023; Kgomotso et al., 2024). This evidence shifts attention toward identifying patients most likely to benefit from stabilization procedures. Accurate assessment of segmental instability therefore becomes central, as it guides decisions regarding the extent of surgical intervention (Elmose et al., 2023; Cabrera et al., 2024; Simmonds et al., 2015).

**Fig. 1:**
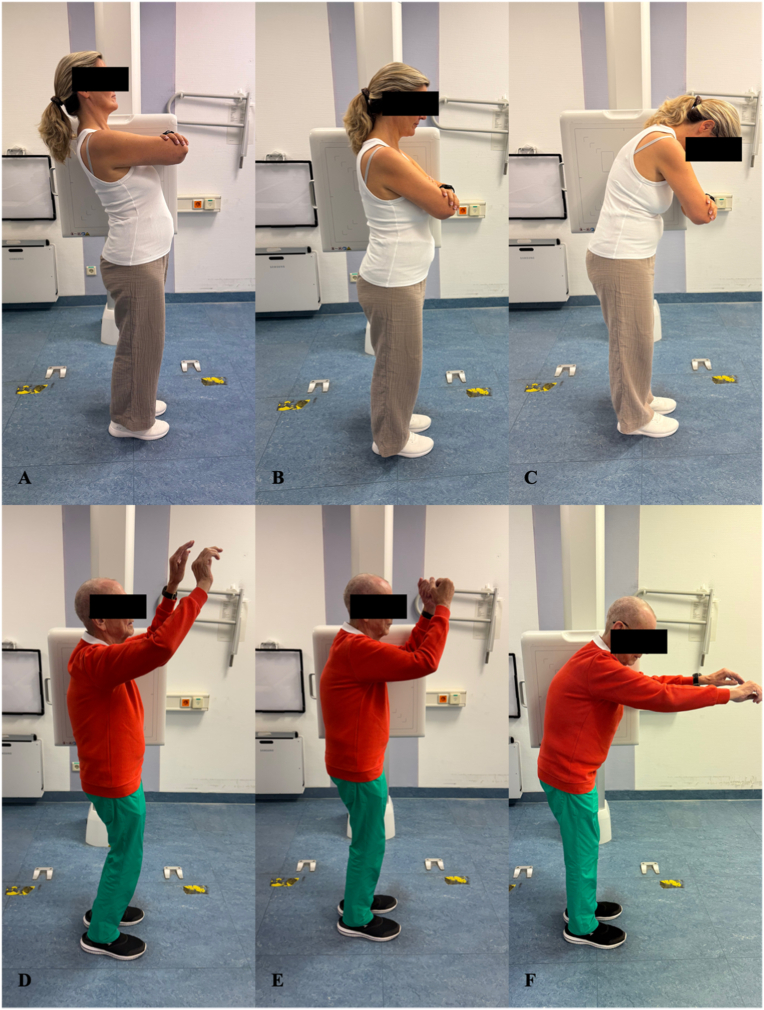
Impact of lumbar mobility on flexion-extension radiographs. Neutral (B, E), flexion (C, F), and extension (A, D) lateral positioning during flexion-extension radiographs in two patients with preserved (A-C) versus impaired (D-F) lumbar mobility. Limited lumbar motion in the second patient highlights the difficulty of performing dynamic flexion-extension imaging in cases of restricted lumbar flexibility.

Radiographic assessment of segmental instability commonly defines pathological motion as sagittal translation of ≥8% of the inferior vertebral body width and/or a dynamic slip angle (SA) of at least 10° (Elmose et al., 2023; Liu et al., 2015; Boden and Wiesel, 1990; Kanemura et al., 2009). Although flexion-extension (FE) radiographs remain the most frequently used modality for assessing segmental mobility in DLS, their use is associated with increased radiation exposure and higher healthcare costs. Moreover, limited reproducibility and restricted applicability in patients with pain or reduced mobility may lead to an underestimation of instability, thereby affecting surgical decision-making (Fig. 1) (Cabrera et al., 2024; Liu et al., 2015; Kashigar et al., 2021; Schroeder et al., 2015).

A less invasive approach to detecting segmental instability without reliance on additional dynamic imaging involves the combination of supine magnetic resonance imaging (MRI) and upright neutral lateral radiographs. Both imaging modalities are typically obtained as part of the standard preoperative diagnostic workup. However, despite prior studies suggesting that this combination may be equivalent or even superior to FE radiographs in detecting lumbar instability, evidence remains inconsistent due to heterogeneous patient populations and varying definitions of instability, leaving its clinical validity uncertain (Elmose et al., 2023; Liu et al., 2015; Kashigar et al., 2021; Chan et al., 2019; Tarpada et al., 2018; Cabraja et al., 2012; Fujimoto et al., 2023).

Therefore, the present study analyzes the largest known cohort of patients with DLS at the L4/5 level to date, with the aim of comparing the degree of segmental motion captured by upright-supine (US) imaging and FE radiography. Furthermore, the investigation evaluates the ability of each modality to detect instability and explores clinical and radiographic factors associated with increased segmental motion.

## 2 Materials and methods

### 2.1 Study design

This retrospective study was approved by the hospital's institutional review board (EA2/194/22). Individual informed consent was waived due to the retrospective nature of the study. The investigation was conducted in accordance with the ethical principles of the Helsinki Declaration (World Medical Association, 2013) and the Strengthening the Reporting of Observational Studies in Epidemiology (STROBE) guidelines (Cuschieri, 2019) were followed in the reporting of this study.

### 2.2 Data collection and population

**Fig. 2:**
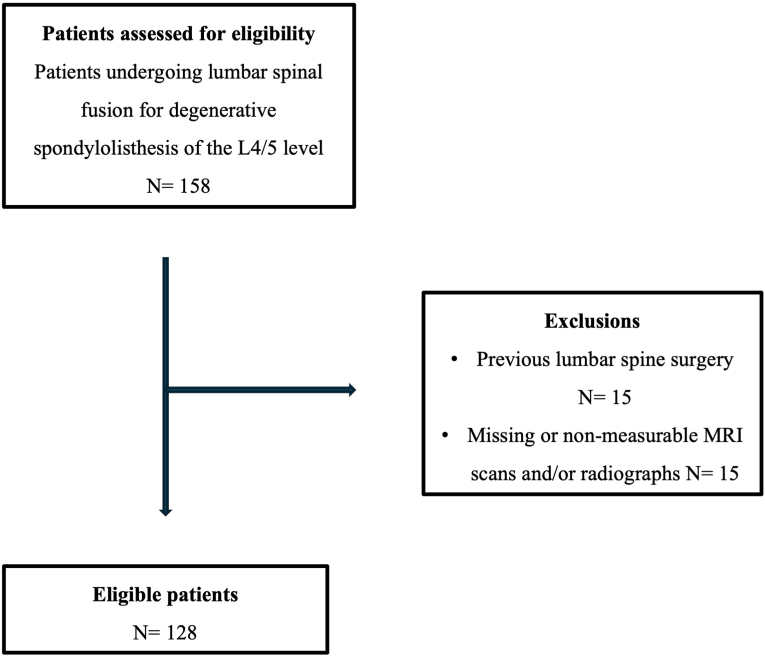
Flow chart of patient inclusion.

A retrospective review was performed using the hospital's medical records system to identify patients who underwent posterior fusion for DLS of the L4/5 level between January 2010 and May 2025. To minimize selection bias, only consecutive patients meeting all inclusion criteria were enrolled. Exclusion criteria were patient age <18 years, any previous lumbar spine surgery, DLS at levels other than L4/5, and imaging datasets of insufficient quality due to missing data, motion artifacts, or incomplete depiction of the L4/5 segment. A detailed overview of patient inclusion and exclusion is provided in the study flowchart (Fig. 2). Patient demographics including age, sex, height, weight, body mass index (BMI), comorbidities and American Society of Anesthesiology (ASA) score (Daabiss, 2011) were retrieved from the electronic patient records.

### 2.3 Definitions and measurement of segmental instability

**Fig. 3:**
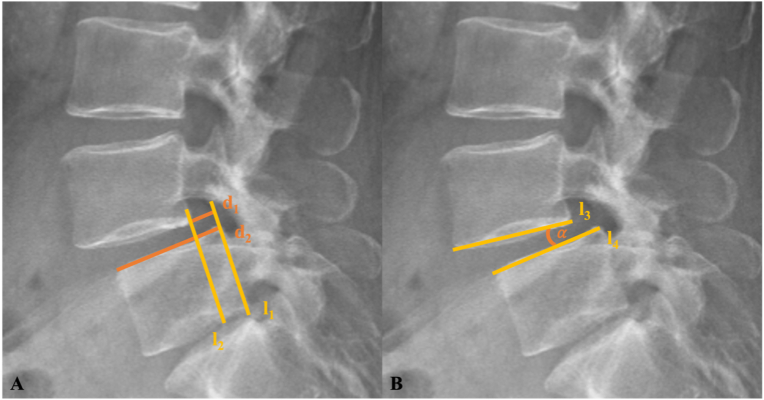
Measurement of Sagittal Slippage (A) (World Medical Association, 2013) and Slip Angle *α* (B) (Cuschieri, 2019), A: Sagittal slippage (d_1_) between parallel lines l_1_ (posterior margin of L5) and l_2_ (posterior inferior corner of L4). Vertebral body width (d_2_) was measured along the superior endplate of L5. B: Slip angle (α) between lines l_3_ (inferior endplate of L4) and l_4_ (superior endplate of L5).

Following the definition by Boden and Wiesel (1990), a sagittal slippage ≥8% of the width of the inferior adjacent vertebral body between flexion and extension was used as the cutoff for segmental instability. The absolute sagittal slippage (d_1_) was determined by drawing a reference line (l_1_) along the posterior margin of the L5 vertebral body (Fig. 3). A second line (l_2_), parallel to l_1_, was drawn to the posterior inferior corner of the L4 vertebral body (Fig. 3). The distance between these two lines represented d_1_, measured in millimeters. To account for interindividual differences in vertebral body size, the anteroposterior length of the superior endplate of L5 (d_2_) was measured (Fig. 3). The relative slippage (RS) of the L4/5 segment was then calculated as the ratio of d_1_ to d_2_, according to the following formula (Liu et al., 2015; Köhli et al., 2024):

I Relative Slippage (RS) [%] = (d_1_ [mm] / d_2_ [mm]) × 100%.

Second, a dynamic SA of at least 10° was employed as an additional indicator of instability, consistent with thresholds established in prior investigations (Elmose et al., 2023; Kanemura et al., 2009). The intervertebral SA (α) was determined by drawing lines along the inferior endplate of the upper vertebra (l_3_) and the superior endplate of the lower vertebra (l_4_) at the affected segment (Fig. 3). The angle formed between these lines represents segmental alignment, with positive values indicating kyphosis and negative values indicating lordosis (Luk et al., 2003).

**Fig. 4:**
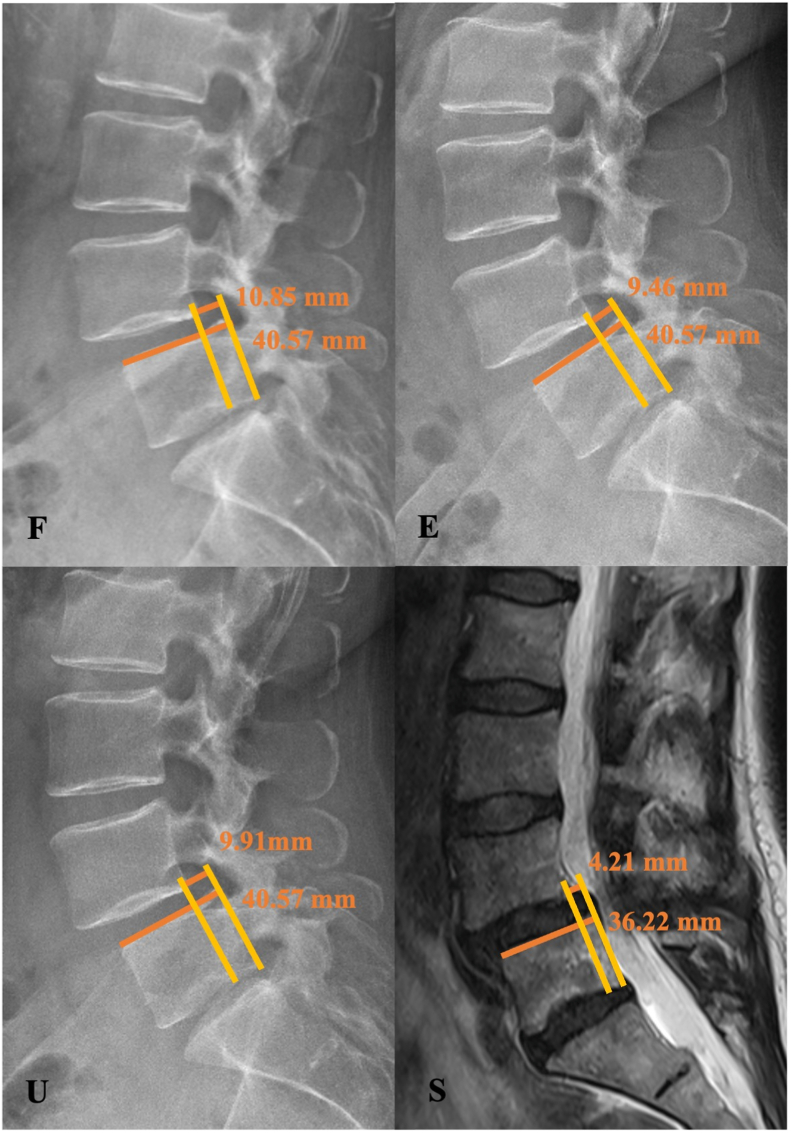
Sagittal translation in a 53-year-old patient with degenerative spondylolisthesis, demonstrating position-dependent variation in segmental motion across flexion, extension, upright, and supine imaging.

Both parameters were assessed using lateral radiographs in flexion (F) and extension (E), upright neutral lateral radiographs (U), and supine-position MRI (S), to quantify position-dependent variation in segmental motion (Fig. 4). MRI was performed in supine position on 1.5-T or 3-T scanners using institutional standard lumbar spine protocols. Axial and sagittal T2-weighted sequences (3–4 mm slice thickness) were obtained. For MRI measurements, the midsagittal slice was selected based on the geometric center of the affected vertebral body and spinal canal identified on axial images, with sagittal images aligned parallel to the vertebral endplates. Upright radiographs were acquired in standing position with knees extended and weight evenly distributed. Neutral lateral views were obtained in relaxed posture with anterior arm support, and flexion-extension views were performed with patients bending forward and backward as tolerated without assistance. As part of the institutional preoperative standard, all imaging studies were completed within three months prior to surgery, ensuring the interval between MRI and radiographs did not exceed three months.

RS and SA were measured for each position, and dynamic changes (ΔRS and ΔSA) were calculated between flexion–extension (F–E) and upright–supine (U–S) imaging. In the present study, segmental instability was therefore defined as either a ΔRS of ≥8% and/or a ΔSA of ≥10°, measured between F to E or U to S (Elmose et al., 2023; Liu et al., 2015; Boden and Wiesel, 1990; Kanemura et al., 2009; Folkerts et al., 2025).

All measurements were performed digitally using the institutional PACS software (Phönix-PACS GmbH, Freiburg im Breisgau, Germany) with integrated DICOM calibration tools. Distances and angles were assessed using standardized line and angle measurement functions, and relative slippage was calculated to reduce magnification-related bias. All images were initially evaluated by one spine surgeon. For reliability analysis, a second independent spine surgeon re-measured a random subset of approximately 30% of cases (39/128). Interrater reliability was assessed using the intraclass correlation coefficient. In cases of substantial discrepancy, consensus review was performed.

### 2.4 Statistical analysis

A formal sample size estimation was conducted for the primary endpoint using a two-sided paired *t*-test framework. The expected effect size was based on the prospective cohort study by Liu et al., which reported a mean difference of approximately 2.8 percentage points between modalities (Liu et al., 2015). To account for potential inter-study variability, a conservative mean difference of 2.0 percentage points was assumed. As prior studies did not consistently report the standard deviation of paired differences, variability was estimated using the observed within-patient SD of paired differences in our cohort (6.50 percentage points). With α = 0.05 and 90% power, the minimum required sample size was 114 patient pairs. The final analyzed cohort exceeded this threshold.

Continuous variables were tested for normal distribution using the Shapiro–Wilk test and are presented as mean ± standard deviation (SD). Categorical variables are reported as absolute numbers and percentages. For comparisons of continuous variables between independent groups, unpaired t-tests were used. Categorical variables were compared using the Chi-square test (χ^2^-test). Within-patient comparisons between imaging modalities were performed using two-sided paired t-tests. Mean paired differences with 95% confidence intervals (CI) were calculated. Effect sizes for paired comparisons were quantified using Cohen's dz. Comparisons of paired binary outcomes were conducted using the McNemar test. Interrater reliability was assessed using a two-way random-effects intraclass correlation coefficient (ICC (Matz et al., 2014; Saremi et al., 2024)), and intrarater reliability using a two-way mixed-effects model (ICC (Elmose et al., 2023; Saremi et al., 2024)).

A multiple linear regression analysis was conducted to examine the predictive value of demographic factors (sex, age, and BMI) on segmental motion assessed in both FE and US imaging. The dependent variables were RS and slip angle. Multicollinearity was assessed using the variance inflation factor (VIF), and residual independence was evaluated using the Durbin-Watson statistic. A VIF <5 and a Durbin-Watson statistic between 1.5 and 2.5 were considered acceptable. Residuals were tested for normal distribution and homoscedasticity. Statistical significance was defined as a p-value <0.05. All statistical analyses were performed using R (Version 4.4.2, R Foundation for Statistical Computing, Vienna, Austria).

## 3 Results

### 3.1 Clinical parameters

A total of 158 patients undergoing lumbar spinal surgery of only the L4/5 level for DLS were identified. After excluding 30 individuals due to previous spine surgery and missing or non-measurable image data, 128 patients remained eligible for analysis. Of these, 81 (63.3%) were female. Patient demographics, ASA scores (Daabiss, 2011), and Meyerding grades (Putzier et al., 2024) stratified by sex are summarized in Table 1. Female patients exhibited a significantly higher frequency of spondylolisthesis Meyerding grades >1 (p = 0.007).

**Table 1:** Clinical parameters (Patient demographics, ASA Score, Meyerding Grade) stratified by sex.

Parameter	All (n = 128)	Female (n = 81)	Male (n = 47)	p-value (f/m)
***Patient demographics***				
Age [years]	69.6 (±10.2)	69.1 (±10.1)	70.3 (±10.5)	0.515
BMI [kg/m^2^]	27.7 (±4.8)	27.5 (±5.1)	28 (±4.4)	0.514
Height [cm]	168.6 (±9.0)	164.0 (±6.6)	176.0 (±7.0)	<0.001
Weight [kg]	78.9 (±16.3)	73.7 (±13.3)	87.8 (±17.2)	<0.001
***ASA Scores***				
I	4/114 (3.5%)	3/73 (4.1%)	1/41 (2.4%)	0.874
II	73/114 (64.0%)	47/73 (64.4%)	26/41 (63.4%)	
III	37/114 (32.5%)	23/73 (31.5%)	14/41 (34.1%)	
***Meyerding Grades***				
1	94 (73.4%)	53 (65.4%)	41 (87.2%)	0.007
2	34 (26.6%)	28 (34.6%)	6 (12.8%)	

### 3.2 Segmental motion

Both interrater and intrarater reliability were excellent for ΔRS and ΔSA measurements, with ICC values exceeding 0.84 for interrater comparisons and 0.90 for intrarater comparisons across all modalities (interrater ICC: ΔRS US 0.92 [0.854–0.957], ΔRS FE 0.863 [0.755–0.925], ΔSA US 0.913 [0.836–0.954], ΔSA FE 0.844 [0.724–0.915]; intrarater ICC: ΔRS US 0.956 [0.918–0.977], ΔRS FE 0.905 [0.827–0.949], ΔSA US 0.957 [0.917–0.978], ΔSA FE 0.93 [0.871–0.962]).

Mean RS differed across imaging modalities. The lowest mean value was observed in the supine MRI position (12.6 ± 6.8%), followed by standing extension radiographs (16.0 ± 8.0%), upright neutral radiographs (20.0 ± 7.7%), and standing flexion radiographs, which showed the highest degree of slippage (23.3 ± 8.5%). ΔRS values measured between US imaging and FE radiography demonstrated comparable values (US: 7.43 ± 5.10%; FE: 7.29 ± 4.57%), with no statistically significant difference observed between the two modalities (p = 0.781; Cohen's dz = 0.025; Table 2).

**Table 2:** Comparison of dynamic changes in relative slippage (ΔRS) and slip angle (ΔSA) between flexion–extension (FE) and upright–supine (US) imaging. Data are presented as mean (SD). Mean differences with 95% confidence intervals (CI) are shown. P-values refer to paired within-patient comparisons using two-sided paired t-tests.

Parameter	Imaging Position		Mean difference (95% CI)	p-value
	Flexion – Extension (FE)	Upright-Supine (US)		
**Δ Relative Slippage [%]**	7.29 (4.57)	7.43 (5.10)	0.14 (−0.98, 1.30)	0.781
**Δ Slip angle [°]**	4.03 (2.98)	6.42 (4.20)	2.39 (1.56, 3.23)	<0.001

Similar to RS, analysis of SA demonstrated position-dependent variation in segmental alignment. Supine MRI demonstrated the most pronounced mean segmental lordosis (−7.05 ± 4.03°), which progressively decreased in standing extension radiographs (−4.57 ± 5.20°) and approximated neutral alignment in upright lateral (−1.00 ± 4.72°) and standing flexion views (−0.96 ± 5.23°). ΔSA was significantly higher for US imaging (ΔSA: 6.42 ± 4.20°) compared with FE radiographs (ΔSA: 4.03 ± 2.98°; p < 0.001; Cohen's dz = 0.501; Table 2).

### 3.3 Segmental instability

**Fig. 5:**
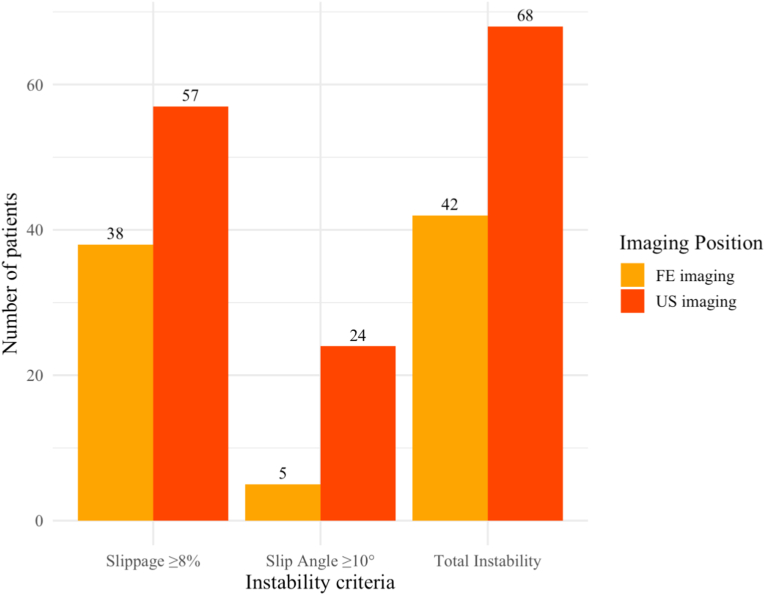
Comparison of radiographically defined instability across FE and US imaging.

US imaging identified a significantly higher number of patients meeting established radiographic thresholds for segmental instability than FE radiography (US: n = 68, 53.1%; FE: n = 42, 32.8%; p < 0.001). These findings are further illustrated in Fig. 5, which summarizes the distribution of radiographically defined instability across imaging positions and diagnostic criteria.

### 3.4 Influence of demographic parameters on segmental motion

Among the assessed demographic variables (sex, age, and BMI), age was the only parameter to exhibit a statistically significant association with segmental motion. In particular, a weak yet statistically significant negative correlation was observed between age and slippage in US imaging (p = 0.028), indicating that older patients exhibited slightly reduced segmental translation in upright radiographs compared to younger individuals. In addition, age was negatively associated with SA in FE imaging (p = 0.004), indicating a statistically significant inverse relationship between patient age and angular motion observed in dynamic FE radiographs. No significant associations were observed for BMI or sex in any of the regression models, and overall model fits (R^2^ ranging from 0.003 to 0.085) indicated that demographic factors explained only a small proportion of the variance in segmental motion.

## 4 Discussion

The present study aimed to compare the extent of segmental motion detected using US imaging versus FE radiographs in patients with DLS at the L4/5 level. ΔRS measurements were comparable between US and FE imaging, whereas ΔSA were significantly higher when assessed using the US approach. Furthermore, US imaging identified a significantly higher number of patients meeting established radiographic thresholds for segmental instability compared to FE imaging.

Despite known limitations, FE radiographs remain the reference imaging modality for evaluating segmental instability to guide surgical decision-making (Cabrera et al., 2024; Kashigar et al., 2021; Schroeder et al., 2015; Morita et al., 2020). A recent cross-sectional survey by Cabrera et al., involving 479 spine surgeons, confirmed that the majority of spine surgeons consider dynamic slippage on FE imaging as the most critical radiographic parameter for determining operative management in the treatment of degenerative spondylolisthesis (Cabrera et al., 2024). The established role of FE imaging is substantiated by its longstanding clinical use and the incorporation of FE-based thresholds into widely accepted definitions of radiographic instability (Elmose et al., 2023; Boden and Wiesel, 1990). However, practical limitations - including restricted patient mobility, pain-limited motion, and increased radiation exposure - have prompted a growing interest in alternative strategies for dynamic assessment (Liu et al., 2015; Kashigar et al., 2021; Jani et al., 2025).

The findings of the present study align with previous investigations suggesting that US imaging may be a sensitive alternative for detecting pathologic segmental motion (Liu et al., 2015; Kashigar et al., 2021; Chan et al., 2019; Viswanathan et al., 2018). In a prospective cohort of 68 patients with DLS and ILS, Liu et al. demonstrated that upright lateral radiographs in combination with supine MRI yielded a higher detection rate of instability compared to FE radiographs (Liu et al., 2015). Similarly, Chan et al. demonstrated in a retrospective analysis of 56 patients with lumbar spondylolisthesis, that dynamic instability was more frequently detected using upright neutral radiographs in combination with supine MRI, compared to FE imaging. Notably, in patients who underwent decompression and fusion, the degree of translation on upright-supine imaging significantly correlated with postoperative improvements in back and leg pain at 12 months (Chan et al., 2019). Further addressing the clinical relevance of instability identified through US imaging, Viswanathan et al. conducted an ambispective study of 77 patients undergoing lumbar fusion, stratified by whether segmental instability was evident on both FE and US imaging or on US imaging alone. Postoperative improvements in pain and disability scores were comparable between both groups, indicating that US imaging may detect clinically meaningful instability not captured by FE radiographs (Viswanathan et al., 2018).

Although several studies highlight the diagnostic utility of US assessment, conflicting evidence suggests that FE radiographs may continue to yield incremental clinical insights (Kashigar et al., 2021; Cabraja et al., 2012; Fujimoto et al., 2023; Thompson et al., 2023). Cabraja et al. retrospectively analyzed imaging of 100 symptomatic patients with low-grade ILS and DLS undergoing lumbar fusion surgery, identifying greater segmental translation on supine computed tomography (CT) compared to upright radiographs than was observed with FE imaging. Nevertheless, 11% of patients demonstrated segmental instability exclusively on FE imaging, which remained undetected on supine or upright radiographs (Cabraja et al., 2012). Similarly, Kashigar et al. assessed the diagnostic yield of FE radiographs in 191 patients with grade I DLS who had already undergone upright radiography and supine MRI. In this cohort, 16% of patients demonstrated additional motion on FE views that was not discernible through comparison of US imaging. Notably, 10% of patients with slips <7 mm on upright radiographs met radiographic thresholds for surgical stabilization only when evaluated with FE radiographs (Kashigar et al., 2021). Furthermore, Fujimoto et al. retrospectively analyzed 92 Japanese patients with DLS to compare segmental mobility between FE radiographs and US imaging. Sagittal translation was significantly greater on FE radiographs, with 17 of 31 patients exhibiting pathological instability in this modality versus ten identified on US imaging. The authors attributed these findings to ethnic differences in spinal alignment and emphasized the necessity of evaluating multiple positions to reliably detect instability (Fujimoto et al., 2023).

Our study adds to this ongoing debate in several important ways. First, to the best of our knowledge, this is the largest single-level analysis to date focusing exclusively on the L4/5 segment - the most frequently affected and surgically treated level in DLS. In contrast to prior studies analyzing multiple spinal levels, we restricted our analysis to a single, clinically predominant motion segment, thereby minimizing intersegmental variability and enhancing clinical applicability of our findings to routine surgical populations. Moreover, by limiting inclusion to symptomatic patients with DLS undergoing surgical treatment, we ensured a homogeneous and clinically pertinent cohort while minimizing confounding from asymptomatic or etiologically distinct cases. Second, we evaluated both RS and SA as markers of segmental instability - an approach that integrates linear and angular displacement to more comprehensively characterize segmental motion. This method addresses a significant limitation of previous studies that utilized one-dimensional metrics. Third, all imaging data were derived from standard supine MRI and upright lateral radiographs acquired as part of routine preoperative evaluation, without additional imaging protocols or patient repositioning. This enhances the clinical feasibility and translational relevance of our approach. Taken together, the structured and methodologically coherent design of our study enhances interpretative validity and helps clarify inconsistencies reported in prior heterogeneous investigations.

The present findings are of considerable clinical relevance, as the role of fusion in DLS remains controversially debated in the current literature (Austevoll et al., 2021; Hellum et al., 2023; Kgomotso et al., 2024; Försth et al., 2016). In particular, the decision between decompression alone and decompression with fusion largely depends on the radiographic assessment of segmental instability (Elmose et al., 2023; Cabrera et al., 2024; Simmonds et al., 2015). Recent randomized trials, including the NORDSTEN study, have demonstrated comparable improvements in disability, pain relief, functional status, and reoperation rates between decompression alone and decompression with fusion, highlighting the importance of refined patient selection rather than routine fusion (Austevoll et al., 2021; Hellum et al., 2023; Kgomotso et al., 2024). In this context, accurate identification of clinically relevant instability remains crucial. While decompression effectively alleviates neurogenic claudication and radicular symptoms, it does not directly address mechanical instability and may exacerbate segmental motion. Fusion aims to restore stability but is associated with increased surgical morbidity, longer recovery, and higher healthcare costs (Cabrera et al., 2024; Reijmer et al., 2024; Morse et al., 2022; Burkhard et al., 2023; Schönnagel et al., 2024). In the present study, US imaging identified higher rates of radiographic instability compared to FE radiographs. However, our study did not assess surgical decision-making or postoperative outcomes, and therefore no conclusions can be drawn regarding the clinical validity of US-only instability findings. Further longitudinal studies are necessary to determine whether US-detected instability reflects clinically meaningful pathology that could enhance surgical decision-making or whether it may lead to an overestimation of instability. At present, US imaging should therefore be interpreted cautiously and in conjunction with FE radiographs, clinical presentation, and additional imaging features suggestive of instability, such as facet joint pathology or soft-tissue indicators on MRI. In cases of discordant findings between imaging modalities, careful clinical correlation is warranted before escalation to fusion surgery. Prospective studies evaluating outcome-based thresholds are required before US imaging can be integrated as an independent determinant of surgical strategy.

This study has several limitations that warrant consideration. First, its retrospective design inherently limits the ability to draw causal inferences and may introduce selection bias. Second, as a single-center study, the external validity of our findings may be constrained by center-specific practices and patient demographics that may not reflect broader clinical populations. Third, although the exclusive focus on the L4/5 level enhances anatomical homogeneity and reduces intersegmental variability, it may limit the generalizability of our findings to other lumbar segments. Furthermore, the exclusive inclusion of patients undergoing fusion for symptomatic L4/5 DLS may have enriched the cohort for suspected instability or advanced pathology, potentially inflating the observed rates of radiographic instability. Consequently, findings may not be generalizable to non-operative or broader DLS populations, and validation in non-surgical cohorts is warranted to clarify the diagnostic utility of US imaging across the full disease spectrum. Additionally, we applied instability thresholds defined for FE radiographs to US imaging. This choice is acknowledged to potentially influence the validity of the comparisons drawn. While this approach permitted direct comparison of modalities, dedicated US-specific thresholds have yet to be established. Future prospective studies are warranted to derive modality-tailored cut-offs. Finally, our study did not assess the impact of imaging findings on surgical decision-making or long-term outcomes. Thus, while US imaging demonstrated higher detection rates of radiographic instability, further validation - including prospective correlation with surgical planning and postoperative results - will be required before its role in routine clinical practice can be firmly established.

## 5 Conclusion

US imaging identified a higher proportion of patients meeting established thresholds for radiographic instability in DLS compared to FE radiographs. Although FE radiography remains the current diagnostic standard, the combination of upright radiographs with supine MRI offers a routinely available, low-radiation alternative that may aid in preoperative assessment. The increased rate of instability detection observed with US imaging highlights its potential diagnostic value; however, the clinical relevance of these additional findings requires further clarification. Future prospective studies should evaluate the predictive validity of US-based instability detection with respect to surgical outcomes and long-term patient benefit.

## Consent for publication

The requirement for individual informed consent was waived by the institutional review board due to the retrospective nature of the study. No identifiable patient data are included in this manuscript.

## Ethical approval

The institutional review board of Charité - Universitätsmedizin Berlin approved this study under the registration number EA2/194/22.

## Authors’ contribution

All listed authors made substantial contribution to the research design, data collection or analysis, and the preparation of this manuscript.

TF: Conceptualization, Methodology, Measurement, Data curation, Formal Analysis, Supervision, Writing-original draft, review and editing.

LS: Data curation, Formal Analysis, review and editing.

LB: Review and editing, Supervision.

LK: Review and editing, Supervision.

TK: Review and editing, Supervision.

MP: Review and editing, Supervision.

FS: Conceptualization, Methodology, Measurement, Data curation, Formal Analysis, Supervision, Visualization, review and editing.

## Data availability statement

The datasets generated and analyzed during the current study are available from the corresponding author upon reasonable request.

## Funding

The authors report no financial support or research grants were received in relation to the planning, execution, or writing of this study.

## Declaration of competing interest

The authors declare that they have no known competing financial interests or personal relationships that could have appeared to influence the work reported in this paper.
